# Identifying Patterns of Tobacco Use and Associated Cardiovascular Disease Risk Through Machine Learning Analysis of Urine Biomarkers

**DOI:** 10.1016/j.jacadv.2025.101630

**Published:** 2025-02-22

**Authors:** Noah A. Siegel, Juan Zhao, Emelia J. Benjamin, Aruni Bhatnagar, Jennifer L. Hall, Andrew C. Stokes

**Affiliations:** a Department of Medicine, Boston Medical Center, Boston University Chobanian, and Avedisian School of Medicine, Boston, Massachusetts, USA; b Data Science and Evaluation, American Heart Association, Dallas, Texas, USA; c Department of Epidemiology, Boston University School of Public Health, Boston, Massachusetts, USA; d American Heart Association Tobacco, Regulation and Addiction Center, University of Louisville School of Medicine, Louisville, Kentucky, USA; e Department of Global Health, Boston University School of Public Health, Boston, Massachusetts, USA

**Keywords:** biomarkers, cardiovascular disease, cluster analysis, electronic cigarettes, machine learning, tobacco phenotype, tobacco use

## Abstract

**Background:**

Tobacco use remains a leading cause of disability-adjusted life years lost in the United States. Cardiovascular harm varies by tobacco product type and usage patterns, yet reliable methods for assessing exposure and harm across different products, especially novel tobacco products, are limited.

**Objectives:**

The authors aimed to identify distinct biomarker exposure patterns associated with different tobacco products using cluster analysis and validate this approach through longitudinal analysis of cardiovascular disease risk.

**Methods:**

Using the Population Assessment of Tobacco and Health data set, we performed cluster analysis and geometric mean modeling of tobacco-related biomarkers, followed by a longitudinal retrospective cohort study with Cox proportional hazard modeling used to examine associations between clusters and a primary composite outcome of heart failure, myocardial infarction, or stroke.

**Results:**

Examining 6,463 individuals, we identified 5 clusters: never users (cluster 1), predominant e-cigarette users (cluster 4), cigarette/dual users (cluster 2), and mixed tobacco users (clusters 3 and 5). All clusters showed elevated biomarkers of oxidative stress and inflammation compared to cluster 1, with clusters 2 and 3 showing the highest levels. Multivariable analysis revealed significantly higher cardiovascular disease risk in cluster 2 vs cluster 1 (HR: 2.24; 95% CI: 1.17-4.30), while other clusters showed elevated but nonsignificant risks.

**Conclusions:**

Our categorization of exposure through cluster analysis provides a potential tool for evaluating the use of emerging tobacco products and establishing a connection between novel exposures and cardiovascular risk. This approach may contribute to the validation of a valuable tool for assessing the risk associated with the use of different tobacco products.

Tobacco use is the second leading risk factor for death and the leading cause of loss of disability-adjusted life years in the United States.^1^ The relationship between cigarette smoke exposure and cardiovascular disease (CVD) risk is nonlinear—risk increases sharply at low exposure levels but rises more gradually as the number of cigarettes smoked per day increases.^2^, ^3^, ^4^ There is a need to quantify individual CVD risk using factors beyond frequency and quantity of tobacco use, as the harm associated with a specific tobacco product depends on both usage patterns and the intrinsic toxicity of product ingredients and constituents. This risk assessment is particularly challenging for novel tobacco products like e-cigarettes, which have unstudied constituent ingredients, unclear long-term health effects, and exist in countless variations.^5^, ^6^, ^7^, ^8^

Biochemical signature profiling has been used in the past to identify characteristics of individuals who use tobacco, such as abstinence.^9^ Previous work has shown that the use of different tobacco products is associated with exposure to chemicals differing from one product to another.^10^ In particular, the use of combustible products such as cigarettes is associated with exposure to high levels of volatile organic compounds (VOCs), poly aromatic hydrocarbons (PAHs), benzopyrenes, metals, and nitrosamines.^11^, ^12^, ^13^, ^14^ Tobacco-specific nitrosamines, such as urinary NNAL and NNNT, have proven useful for assessing tobacco exposure across varied products and differentiating between types of tobacco use.^15^, ^16^, ^17^, ^18^ Studies of e-cigarette users have revealed distinct exposure patterns, with lower urinary nicotine levels compared to traditional cigarette smokers, despite similar nicotine metabolite profiles between groups.^19^ Transition from cigarette to e-cigarette use has been associated with decreases in some biomarker, particularly for compounds like benzene and specific PAHs, though levels of some PAHs remain unchanged.^20^

Some effort has also been made to relate biomarkers of tobacco exposure to clinical outcomes.^21^^,^^22^ While cluster analysis has previously been used to segment tobacco product users based on urinary biomarkers in the Population Assessment of Tobacco and Health (PATH) data set, prior literature lacked the longitudinal follow-up now available through 6 waves of PATH data, which provides insight into the cardiovascular relevance of a clustering approach.^23^

The emergence of novel tobacco products, including heated tobacco devices and oral nicotine pouches, presents a shifting landscape in which traditional survey questions may not fully capture the scope of exposure and associated health risks. While e-cigarettes are likely less harmful than conventional cigarettes for cardiovascular health, emerging evidence from both preclinical and clinical studies suggests they are not harm-free, with documented effects including increased arterial stiffness, oxidative stress, inflammation, and autonomic dysfunction.^24^ The long-term cardiovascular risks of e-cigarette use remain unclear due to their relatively recent introduction to the market.^25^^,^^26^ Though e-cigarettes might serve as a harm reduction tool for current smokers, their cardiovascular impact for individuals initiating tobacco use with e-cigarettes must be weighed.^27^ In this study, we used cluster analysis to identify groups of tobacco users based on their urinary biomarkers. By connecting these objective, biochemically defined clusters to measures of inflammation and oxidation, as well as longitudinal CVD outcome data spanning 8 years of follow-up, we aim to provide novel insights into how specific tobacco exposure patterns relate to CVD risk.

## Methods

The methodology for this study was approved by the Institutional Review Board of Boston Medical Center and Boston University Medical Campus IRB number H-36461. The first author had access to all data used in the study and takes responsibility for its integrity and the data analysis. Data are available from the U.S. Department of Health and Human Services via the Inter-university Consortium for Political and Social Research.^28^ A broad overview of the methodology can be seen in the Central Illustration.

**Central Illustration:**
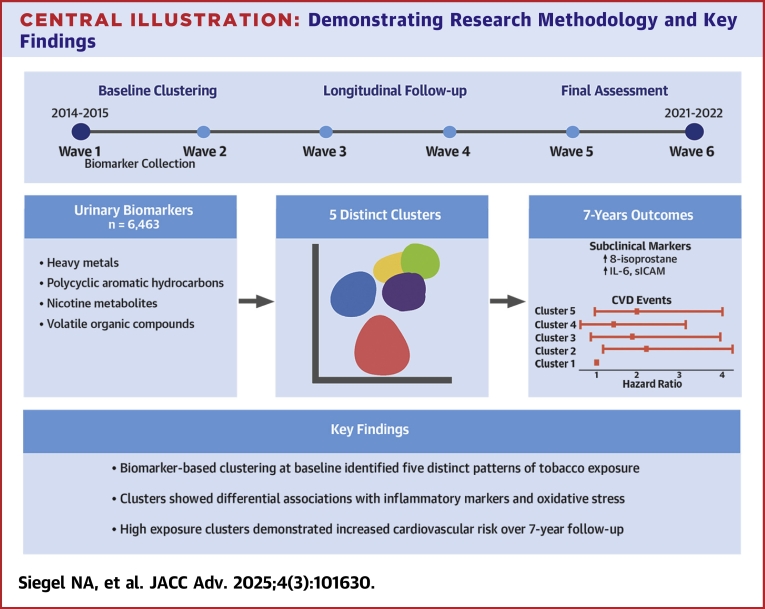
**Demonstrating Research Methodology and Key Findings** CVD = cardiovascular disease; sICAM = soluble intercellular adhesion molecule.

### Participants and eligibility criteria

The PATH Study is a nationally representative, longitudinal cohort study of 45,971 adults and youth in the United States. As shown in Supplemental Figure 1, participants were included in our study if they met the following criteria: 1) were at least 18 years of age, 2) contributed a biomarker sample at wave 1 that was selected for analysis by the PATH study team, 3) used either cigarettes, e-cigarettes, or both at wave 1 of data collection, or had no tobacco product use history, 4) had no history of CVD, and 5) had at least 1 year of follow-up data on CVD status. CVD was defined as self-reported myocardial infarction (MI), heart failure (HF), or stroke. Individuals were excluded if they did not meet all of these criteria. The study utilized 6 waves of PATH study data, accounting for 8 years of follow-up (Supplemental Figure 4). Participants were followed until development of CVD, end of data collection, or loss to follow-up.

Participants were classified into 4 tobacco user types based on their self-reported use patterns: exclusive e-cigarette users, who had ever used an e-cigarette, used fairly regularly, and used every day or some days at the time of data collection, but did not meet criteria for cigarette use; exclusive cigarette users, who had ever smoked a cigarette, smoked more than 100 cigarettes in their lifetime, and smoked every day or some days, but did not meet criteria for e-cigarette use; dual e-cigarette and cigarette users, who met criteria for both e-cigarette and cigarette use; and never users, who had never used any tobacco product, including ones not previously listed.

### Cluster identification

Cluster analysis is a data-driven, unsupervised machine learning technique used to identify groups (clusters) in high-dimensional data sets. This approach has been previously used to analyze potential tobacco use subgroups,^23^ and in other settings, to identify subpopulations across a wide range of diseases.^29^

Our aim for the present study was to identify patterns of tobacco use through cluster analysis and examine the association of these clusters with cardiovascular outcomes. Our cluster analysis was performed using only objective urinary biomarker data, excluding self-reported metrics like frequency and quantity of use. This approach avoided potential conformity bias introduced by participant responses. The analysis was conducted using the Gaussian clustering algorithm in the Python scikit module, version 1.0.2.^30^ Gaussian clustering is well established in the literature, especially with regard to tobacco, and has been applied in numerous studies to identify subpopulations with biomarker profiles.^23^^,^^31^^,^^32^ Gaussian distributions were initialized across the biomarker feature space, and their parameters were iteratively optimized using expectation-maximization until convergence, allowing each data point to be probabilistically assigned to the most likely distribution.

The analysis included all biomarkers listed in Supplemental Table 1, except for biomarkers of potential harm and inflammation (interleukin 6, soluble intercellular adhesion molecule [sICAM], 8-isoprostane, and high-sensitivity C-reactive protein [hsCRP]). These were excluded to minimize potential biases from downstream effects of tobacco use. Demographic and socioeconomic covariates were also excluded for the same reason. Well-validated biomarkers such as urinary NNAL were included in the clustering. Prior to clustering, biomarker values were normalized to urinary creatinine concentration, log-transformed, and missing values were imputed.

We determined the optimal number of clusters using the Bayesian information criterion, calculating values for models with 1 to 11 clusters. Based on these results (Supplemental Table 2), we selected a 5-cluster model as the optimal balance between model fit and parsimony. A 5-cluster model showed the lowest Bayesian information criterion value and additional clusters did not substantially improve model fit.

**Figure 1:**
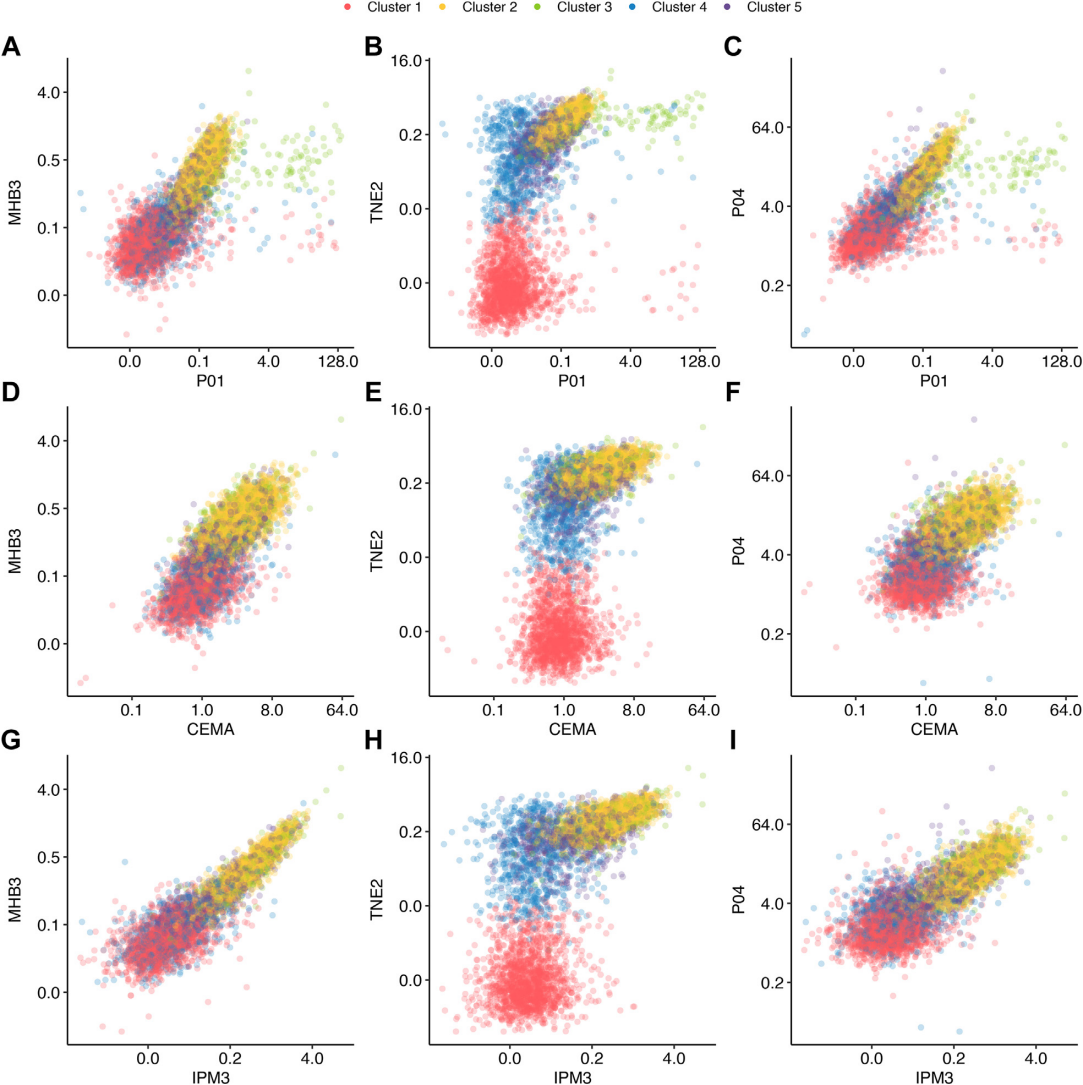
**Distribution of Selected Biomarkers by Cluster Membership** Figure showing distribution of biomarkers by cluster membership. Panels A through I demonstrate that clusters have a variety of shapes with respect to different biomarkers, and show some of the patterns of biomarker distribution. In general Cluster 1 tended to have lower levels of biomarkers. Whereas Cluster 2 had higher levels. Cluster 4 tended to be an intermediate cluster, and then some distributions overlapped with other clusters; Clusters 3 and 5 also occupied an intermediate position. This highlights the benefit of the clustering approach in its ability to elucidate these patterns and identify biomarkers of significance. CEMA = N-acetyl-S-(2-carboxyethyl)-L-cysteine (ng/mL, volatile organic compound); IPM3 = N-acetyl-S-(4-hydroxy-2-methyl-2-buten-1-yl)-L-cysteine (ng/mL, volatile organic compound); MHB3 = N-acetyl-S-(4-hydroxy-2-buten-1-yl)-L-cysteine (ng/mL, volatile organic compound); P01 = 1-naphthol or 1-hydroxynaphthalene (μg/L, polycyclic aromatic hydrocarbon); P04 = -hydroxyfluorene (ng/L, polycyclic aromatic hydrocarbon); TNE2 = trans-3'-hydroxycotinine, urine (nmol/mL, nicotine metabolite).

The resulting clusters are shown in Figure 1. Cluster labels were determined based on the predicted probability of membership by tobacco use pattern, shown in Supplemental Figure 2. These predicted probabilities were obtained using the Stata “margins” command to calculate the probability of cluster membership based on tobacco use patterns from a previously fit logistic regression model. Appropriate survey weighting variables were applied to adjust for the PATH study design.

### Subclinical risk analysis

We selected biomarkers representing subclinical markers of tobacco harm as dependent variables, including inflammatory markers (hsCRP, interleukin-6, fibrinogen, and sICAM) and an oxidative stress marker (urinary 8-isoprostane). To avoid influencing the clustering algorithm, these biomarkers were excluded from the clustering procedure. This decision was made to ensure that our clusters were based solely on exposure biomarkers, rather than markers of potential harm, so that harm could be assessed as a dependent variable. To examine the association between cluster membership and outcome biomarkers, we constructed multivariable linear regression models with sequential covariate adjustments. The coefficients of the multivariable linear models were exponentiated to obtain geometric mean ratios (GMRs). The first model was adjusted for covariates potentially associated with tobacco use and CVD, including age, sex, race, ethnicity, and education. A second model was additionally adjusted for body mass index, hypertension, high cholesterol, diabetes, and family history of MI. We further adjusted analyses for marijuana use history, cigarette pack-years, pack-years squared, and time-varying current use of both noncigarette combustible tobacco products (hookah, pipe, cigar, cigarillo) and noncombustible tobacco products (snus, other smokeless tobacco). Missing data on covariates were imputed using multiple imputations with chained equations. Missing biomarker data were not imputed for postclustering analyses.

### Cardiovascular risk analysis

A longitudinal retrospective cohort study was performed to examine the association between the clusters and CVD. The CVD endpoint used in this study was previously validated in the PATH data set.^33^ Cox proportional hazard analysis was used to estimate the HR for CVD. Participants were followed until the development of CVD, loss to follow-up, or completion of wave 6 of data collection (7 years). Cox models were adjusted to mirror our subclinical risk analysis, with 2 sequential models: the first adjusted for age, sex, race, ethnicity, and education, while the second incorporated the additional covariates detailed in the preceding paragraph. All statistical analyses after cluster generation were conducted using StataMP 17. A 2-sided *P* value of <0.05 was considered statistically significant.

## Results

### Characterization of clusters

The study sample consisted of 6,463 individuals with diverse demographic characteristics (Table 1). The largest group was aged 25 to 34 years, with a predominance of females and individuals identifying as White race. A wide range of educational backgrounds was represented. Individuals aged 18 to 24 years were more likely to belong to cluster 4, while those aged 25 to 34 years were less likely to be in cluster 2 and 3 compared to clusters 1, 4, and 5. Participants aged 65 years and above were more likely to be in cluster 1.

**Table 1:** Demographic Characteristics of Sample

	N (Weighted %)	Cluster 1	Cluster 2	Cluster 3	Cluster 4	Cluster 5
Age, y						
18-24	1,919 (18.4%)	664 (18.6%)	583 (15.6%)	62 (9.41%)	280 (26.1%)	330 (22.7%)
25-34	1,477 (24.6%)	382 (21.5%)	636 (27.3%)	89 (20.8%)	207 (30.4%)	264 (29.4%)
35-44	1,135 (19.2%)	236 (18.7%)	494 (20.2%)	91 (20.8%)	129 (18.4%)	185 (19.3%)
45-54	1,020 (16.9%)	194 (15.5%)	470 (19.1%)	110 (24.2%)	93 (14.8%)	153 (16.0%)
55-64	623 (11.9%)	134 (12.5%)	292 (12.6%)	72 (17.0%)	47 (6.82%)	78 (8.94%)
65-74	227 (5.82%)	76 (7.72%)	82 (4.36%)	29 (6.64%)	15 (2.27%)	25 (2.89%)
75+	62 (2.91%)	34 (5.19%)	12 (0.50%)	3 (0.96%)	8 (0.95%)	5 (0.55%)
Sex						
Male	3,167 (47.0%)	641 (38.4%)	1,317 (55.2%)	224 (53.2%)	413 (53.7%)	572 (59.3%)
Female	3,293 (52.9%)	977 (61.5%)	1,251 (44.7%)	232 (46.7%)	366 (46.2%)	467 (40.6%)
Ethnicity						
Hispanic	1,039 (15.9%)	453 (21.8%)	227 (7.39%)	42 (8.69%)	163 (16.8%)	154 (11.5%)
Not Hispanic	5,357 (84.0%)	1,154 (78.1%)	2,318 (92.6%)	402 (91.3%)	608 (83.1%)	875 (88.4%)
Race						
White	4,655 (74.6%)	1,065 (71.2%)	2,056 (83.8%)	333 (77.3%)	570 (77.4%)	631 (64.2%)
Black	1,015 (14.5%)	297 (14.7%)	259 (9.85%)	87 (17.5%)	102 (13.6%)	270 (24.8%)
Other, including multiple	668 (10.8%)	201 (14.0%)	227 (6.31%)	30 (5.09%)	85 (8.91%)	125 (10.8%)
Education						
Less than high school	1,630 (20.7%)	273 (15.5%)	771 (27.6%)	136 (29.3%)	172 (20.9%)	278 (24.0%)
High school graduate	1,608 (25.8%)	393 (24.1%)	675 (29.3%)	104 (25.8%)	180 (24.0%)	256 (27.2%)
Some college	2,415 (33.1%)	573 (29.7%)	936 (35.2%)	179 (36.4%)	315 (39.8%)	412 (37.6%)
Bachelor's degree	548 (13.0%)	229 (18.2%)	138 (6.17%)	27 (6.85%)	83 (12.0%)	71 (9.18%)
Advanced degree	240 (7.11%)	142 (12.3%)	53 (1.62%)	9 (1.46%)	24 (3.12%)	22 (1.93%)

This table displays demographic characteristics of the study population, including age, sex, ethnicity, race, and education. Differences are seen in demographic composition of the clusters consistent with national patterns when considering cluster tobacco use patterns.

Predictive probabilities of cluster membership by tobacco use pattern were informative in assigning meaning to clusters. As shown in Supplemental Figure 2, cluster 1 was characterized by individuals who never used tobacco products, effectively serving as a reference cluster. Never users demonstrated a high probability (0.96) of assignment to cluster 1. Cluster 4 was predominantly associated with e-cigarette use, with individuals using e-cigarettes having a 0.64 probability of assignment to this cluster. This cluster also included some individuals who used cigarettes (0.11 probability) and dual users of both products (0.15 probability). Cluster 2 was predominantly associated with cigarette use (0.52 probability) and dual use (0.45 probability). Cluster 3 and cluster 5 showed more mixed probabilities across user types, with cluster 5 having high probabilities for cigarette users (0.21) though ultimately higher for and dual users (0.25). This pattern was consistent with cluster 3 as well. The probabilistic nature of the clustering approach allowed for a more nuanced representation of tobacco use patterns, capturing the complex relationships between different user types across clusters.

### Cluster biomarker patterns

**Figure 2:**
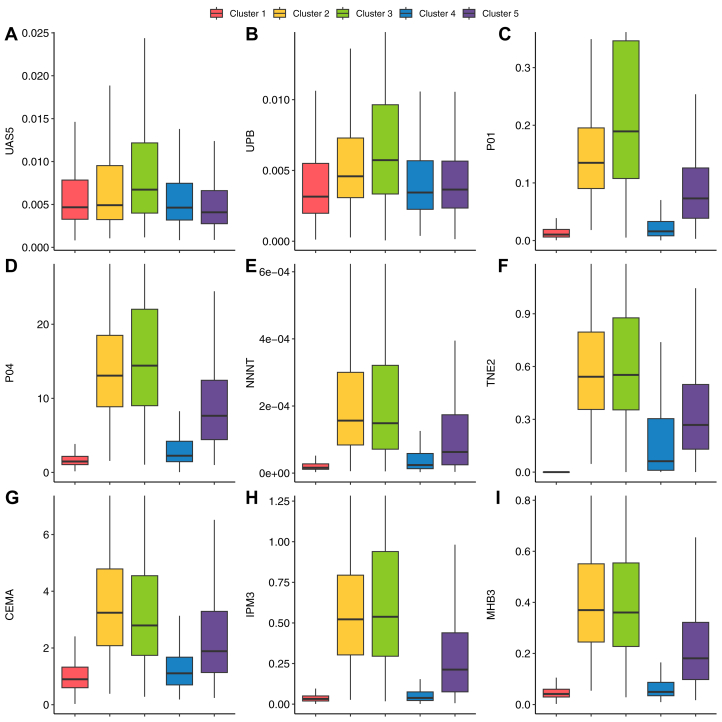
**Selected Biomarker Comparison by Cluster** Panels A through I showing distribution of individual selected biomarkers by cluster. Some biomarkers such as Arsenic acid, urine (μg/L), Lead, urine (μg/L) (UAS5, UPB: Heavy metals) were relatively uniform between clusters. Whereas other biomarkers like Trans-3'-Hydroxycotinine, urine (nmol/mL), N-Acetyl-S-(4-hydroxy-2-methyl-2-buten-1-yl)-L-cysteine (ng/mL) (TNE2, IPM3: Nicotine metabolite and Volatile organic compound, respectively) showed extreme variation between clusters. This is suggestive of the intrinsic differences between clusters and features defining them. CEMA = N-acetyl-S-(2-carboxyethyl)-L-cysteine (ng/mL, volatile organic compound); MHB3 = N-acetyl-S-(4-hydroxy-2-buten-1-yl)-L-cysteine (ng/mL, volatile organic compound); NNNT = N'-nitrosonornicotine (ng/mL, nicotine metabolite); P01 = 1-naphthol or 1-hydroxynaphthalene (μg/L, polycyclic aromatic hydrocarbon); P04 = 2-hydroxyfluorene (ng/L, polycyclic aromatic hydrocarbon).

Broadly speaking, the 5 clusters exhibited distinct biomarker profiles, as illustrated in Figure 2 and Supplemental Figure 3. Cluster 1 generally had the lowest levels of biomarkers of tobacco exposure, which is expected given the high prevalence of nonusers of tobacco in cluster 1. Cluster 4 had intermediate to low levels of biomarkers of tobacco exposure, with higher levels of most biomarkers of tobacco exposure, however decreased from clusters 2, 3, and 5. Clusters 2, 3, and 5 showed the highest overall biomarker levels, with clusters 2 and 3 differing primarily in key VOCs and heavy metals. Cluster 5 exhibited lower overall levels compared to clusters 2 and 3. Urinary levels of heavy metals, including lead and arsenic, showed relatively consistent levels across all clusters. The multidimensional nature of the clustering is evident in Figure 1, which illustrates the complex relationships between selected biomarkers across the 5 clusters. Clusters 1 and 4 had significant overlap among certain biomarkers of tobacco exposure, while maintaining distinct profiles of other biomarkers as can be seen in Figure 2.

### Subclinical markers of harm

We calculated the geometric means and GMRs of relevant subclinical markers of harm as previously described. As shown in Table 2, compared to cluster 1, all other clusters demonstrated elevated levels of urinary 8-isoprostane, a biomarker of lipid peroxidation. Cluster 3 exhibited the highest levels with a GMR of 1.62 (95% CI: 1.52-1.72), followed by cluster 2 (1.48; 95% CI: 1.41-1.54), cluster 5 (1.36; 95% CI: 1.29-1.43), and cluster 4 (1.19; 95% CI: 1.13-1.25). Similarly, in multivariable-adjusted models, urinary levels of the cytokine IL-6 were significantly higher in clusters 2, 3, and 5 compared to cluster 1, with GMRs of 1.16 (95% CI: 1.08, 1.24), 1.25 (95% CI: 1.13, 1.38), and 1.16 (95% CI: 1.07, 1.25), respectively. The inflammatory marker sICAM showed a similar pattern, with all clusters except cluster 4 demonstrating significantly higher levels than cluster 1. The levels of the inflammatory marker hsCRP were significantly higher only in cluster 2 compared to cluster 1 (1.18; 95% CI: 1.05-1.32). These associations persisted after adjusting for baseline covariates potentially associated with tobacco use and CVD. Notably, cluster 3 consistently showed the highest levels of inflammatory and oxidative stress markers across all measured indicators, while cluster 4 had the weakest association with elevated inflammatory and oxidative stress markers.

**Table 2:** Geometric Mean Ratios of Biomarkers of Inflammation and Oxidation by Cluster

	Cluster 1	Compared With Cluster 1			
		Cluster 2	Cluster 3	Cluster 4	Cluster 5
8-isoprostane					
Adjusted geometric mean	3.37 (3.13-3.63)				
Age-, sex-, race-/ethnicity-adjusted GMR (95% CI)	Ref	1.58 (1.52-1.64)	1.75 (1.65-1.85)	1.25 (1.19-1.31)	1.43 (1.37-1.50)
Multivariable-adjusted GMR (95% CI)	Ref	1.48 (1.41-1.54)	1.62 (1.52-1.72)	1.19 (1.13-1.25)	1.36 (1.29-1.43)
High-sensitivity C-reactive protein					
Adjusted geometric mean	0.26 (0.21-0.32)				
Age-, sex-, race-/ethnicity-adjusted GMR (95% CI)	Ref	1.17 (1.05-1.31)	1.15 (0.97-1.37)	1.04 (0.91-1.20)	1.11 (0.97-1.27)
Multivariable-adjusted GMR (95% CI)	Ref	1.18 (1.05-1.32)	1.15 (0.97-1.36)	0.99 (0.86-1.13)	1.07 (0.94-1.22)
Interleukin 6					
Adjusted geometric mean	0.48 (0.43-0.54)				
Age-, sex-, race-/ethnicity-adjusted GMR (95% CI)	Ref	1.17 (1.10-1.24)	1.27 (1.15-1.40)	1.07 (0.98-1.15)	1.18 (1.10-1.27)
Multivariable-adjusted GMR (95% CI)	Ref	1.16 (1.08-1.24)	1.25 (1.13-1.38)	1.03 (0.95-1.12)	1.16 (1.07-1.25)
Soluble intercellular adhesion module					
Adjusted geometric mean	190.0 (178.5-202.3)				
Age-, sex-, race-/ethnicity-adjusted GMR (95% CI)	Ref	1.24 (1.20-1.28)	1.28 (1.21-1.34)	1.05 (1.01-1.09)	1.15 (1.10-1.19)
Multivariable-adjusted GMR (95% CI)	Ref	1.22 (1.18-1.27)	1.25 (1.18-1.32)	1.04 (1.00-1.09)	1.14 (1.09-1.19)

This table compares adjusted geometric mean ratios (GMRs) of biomarkers of harm among distinct clusters of tobacco product users and a reference group. Biomarkers including 8-isoprostane, high-sensitivity C-reactive protein, interleukin 6, and soluble intercellular adhesion molecule were assessed. Clusters 2 and 3 (cigarette and dual use associated) generally show the highest elevations across biomarkers. Cluster 4 (e-cigarette associated) demonstrated elevated biomarkers of subclinical harm, although consistently less than others when compared to cluster 1. Multivariable adjustment consisted of adjusting for body mass index, hypertension, high cholesterol, diabetes, and family history of MI. Analyses were also adjusted for ever use of marijuana, cigarette pack-years, and pack-years squared. Time-varying current use of noncigarette combustible (hookah, pipe, cigar, cigarillo) and noncombustible (snus, other smokeless) tobacco were also included.

### Incident CVD

A retrospective cohort study examined the association between the 5 identified clusters and the incidence of CVD, specifically MI, HF, or stroke, over a 7-year follow-up period from October 2014 to November 2021. A total of 326 CVD events were documented during the follow-up period, with the mean age at the time of these events being 57.4 years. As shown in Table 3, the incidence rates of CVD events varied across clusters, with cluster 1 showing the lowest rate at 1.86 per 1,000 person-years (95% CI: 0.93-2.79) and cluster 2 the highest at 4.20 per 1,000 person-years (95% CI: 2.94-5.46). In multivariable analysis adjusted for age, sex, race, ethnicity, education, income, and smoking status, individuals in cluster 2 had the highest risk of CVD compared to those in cluster 1 (reference group), with a HR of 2.24 (95% CI: 1.17-4.30). Clusters 3, 4, and 5 also showed elevated risks compared to cluster 1, with HRs of 1.89 (95% CI: 0.89-3.99), 1.45 (95% CI: 0.66-3.17), and 1.99 (95% CI: 0.98-4.05), respectively. However, in the fully adjusted model, only cluster 2's increased risk achieved statistical significance. These findings suggest differential CVD risk associations across clusters, with cluster 2 demonstrating the most pronounced and statistically significant elevation in risk compared to the reference cluster 1.

**Table 3:** Multivariable Adjusted Hazard of MI, HF, or Stroke Over 6 Years

	Cluster 1	Compared With Cluster 1			
		Cluster 2	Cluster 3	Cluster 4	Cluster 5
Myocardial infarction, heart failure, or stroke					
Incidence rate, per 1,000 person-years (95% CI)	1.86 (0.93-2.79)	4.20 (2.94-5.46)	3.52 (1.76-5.28)	2.69 (1.17-4.21)	3.73 (2.03-5.43)
Age-, sex-, race- and ethnicity-, education- adjusted HR (95% CI)	Ref	2.45 (1.39-4.34)	2.06 (1.04-4.09)	1.43 (0.69-2.99)	2.13 (1.10-4.10)
Multivariable-adjusted HR (95% CI)	Ref	2.24 (1.17-4.30)	1.89 (0.89-3.99)	1.45 (0.66-3.17)	1.99 (0.98-4.05)

Table showing incidence of MI, HF, or stroke for 5 waves (7 years) after clustering. Cluster 2 (cigarette use associated) had increased incidence of multivariable adjusted MI, HF, or stroke when compared with a reference group. Multivariable adjustment consisted of adjusting for body mass index, hypertension, high cholesterol, diabetes, and family history of MI. Analyses were also adjusted for ever use of marijuana, cigarette pack-years, and pack-years squared. Time-varying current use of noncigarette combustible (hookah, pipe, cigar, cigarillo) and noncombustible (snus, other smokeless) tobacco were also included.

HF = heart failure; MI = myocardial infarction.

## Discussion

The major findings of our study are that cluster analysis identified distinct subgroups of individuals based on their exposures to tobacco-derived chemicals using objective urinary data alone. These identified clusters aligned with specific self-reported tobacco product use patterns, correlated with biomarkers of harm, and corresponded to differences in cardiovascular risk over 7 years of follow-up.

The pattern of exposure to high levels of harmful or potentially harmful chemicals (HPHCs) was associated with use of combustible cigarettes or dual use and was distinctly different from the cluster which did not use tobacco products (cluster 1). A large group of individuals showed intermediate levels of exposure (cluster 4) and was associated with individuals who exclusively used e-cigarettes (Supplemental Figure 2). Cluster 4 likely includes individuals with low exposure levels either due to infrequent use of combustible cigarettes or use of products with lower HPHC emissions. The higher levels of nicotine metabolites in clusters 2, 3, 5, and to a lesser degree, 4, than cluster 1 suggest greater frequency of use of nicotine-containing products by individuals in those clusters.^34^ The demographic differences between clusters were relatively correlated with national distributions of tobacco using groups.^35^ The geometric mean modeling also produces similar results when considering national patterns.^36^ Taken together, these results suggest that clustering on the basis of the levels of HPHC exposure could be a useful approach to identify use patterns and map them on to the use of specific tobacco products. Cluster analysis may be equivalent to, or better, at capturing real-world use patterns than the self-reported questions on tobacco use.

Cluster analysis could also be used to link exposure to outcomes. Individuals with higher HPHC exposure (clusters 2, 3, and 5) had higher levels of biomarkers of oxidative stress and inflammation than those with low levels of exposure (cluster 1), consistent with the notion that exposure to tobacco-derived chemicals could induce chronic oxidative stress. Prior work has shown that PAH and VOC exposure may associate with or contribute to the development of CVD in smokers and nonsmokers.^37^, ^38^, ^39^, ^40^

Notably, levels of the biomarkers of harm were higher in individuals with high levels of exposure (cluster 2) than those with intermediate levels of exposure (cluster 4), suggesting a dose-dependent relationship between exposure and inflammation. Overall, the results of these analyses suggest that the tobacco product use pattern in cluster 2 is associated with significantly higher levels of oxidative stress and inflammation compared to nonuse. In our previous work with the Multi-Ethnic Study of Atherosclerosis cohort, we reported that smoking intensity was associated with early biomarkers of CVD, consistent with the view that higher smoking rates, and thereby higher levels of exposure to HPHCs, are associated with greater changes in biomarkers of harm.^41^ These biomarkers of harm are in turn directly related or indirectly related to development of CVD morbidity and mortality.^42^, ^43^, ^44^, ^45^ In this sense, clustering of urinary biomarkers is able to serve as an objective “link” between use patterns and markers of subclinical oxidation and inflammation.

Despite the clear dose dependence of biomarkers of harm with exposure, the relationship between HPHC exposure and CVD outcomes was less clear. Analysis of specific cardiovascular outcomes—MI, HF, or stroke—showed that clusters 2, 3, and 5 had significantly higher risk than cluster 1. This risk was attenuated with further model adjustment, with only cluster 2 maintaining a significant association. Cluster 4 showed an elevated but statistically insignificant risk. This finding warrants careful interpretation, particularly in light of potential confounding factors. That clusters with large differences in exposure may lead to similar risks is surprising but may relate to the highly nonlinear relationship between smoking and cardiovascular outcomes.^46^

Detailed analysis of the dose response curve shows that the CVD risk associated with 2 pack of cigarettes a day is only 20% higher than smoking 3 cigarettes a day,^47^ indicating that even low levels of exposure (eg, due to low smoking intensity, low HPHC device, or indirect exposure to secondhand smoke) may be associated with significant CVD risk. The highly nonlinear relationship between tobacco product use and CVD may be significant in assessing the effects of e-cigarettes in which reduction in toxicant exposure has been assumed to lead to proportional reduction in harm.

Our study has several strengths. Its findings are based on data from a large longitudinal study of a nationally representative population, with comprehensive evaluation of tobacco product use history and assessments of a wide range of biomarkers. These findings provide new insights into the patterns of tobacco use biomarkers among individuals, and the potential to use these biomarkers to identify clinically relevant characteristics. Using only a urine sample, clustering was able to recreate cohorts of individuals who use e-cigarettes and cigarettes. Clustering may be an effective way to identify individuals who use e-cigarettes and cigarettes without relying on self-reported survey questionnaires. Compared to well-validated long-persisting tobacco-specific biomarkers like NNAL, cluster analysis provides a multidimensional method of assessing new and emerging tobacco products. The strength of unsupervised learning techniques is seen in clustering analysis with emergent patterns that may not be fully captured with traditional epidemiological methods. Additionally, with more specific biomarkers, clustering may be a valid way to ascertain more specific phenotypes of use and predict clinical harm.

### Study Limitations

The limited timeframe to develop cardiovascular events may not fully capture the long-term impact of tobacco use on health outcomes. Further research with larger sample sizes and longer follow-up periods is needed to confirm and expand upon our findings. Cardiovascular events were self-reported which may lead to misclassification, though our previously validated analysis using only HF, MI, and stroke seeks to mitigate that concern. A significant limitation is that our cluster analysis was based on tobacco use patterns from 2014, when e-cigarette devices were substantially different from current products. The identified clusters may not accurately represent contemporary use patterns, necessitating updated analyses for current and future prediction purposes. In prior studies, tobacco use patterns showed considerable variation throughout the PATH follow-up period, with documented transitions between cigarette use, e-cigarette use, and dual use.^48^^,^^49^ Therefore, the baseline clusters may not reflect participants' actual exposure patterns during the follow-up period. This was partially mitigated by adjusting analyses for time varying use of noncigarette combustible and noncombustible (non e-cigarette) tobacco.

The average age of participants who developed CVD was 57 years. Given this age profile, most e-cigarette users in our study would have been former cigarette smokers, making it difficult to distinguish between the cardiovascular effects of e-cigarette use and residual risk from previous smoking history. While we controlled for pack-years in our analysis, this measure may not fully capture the nuances of participants' smoking histories, including the timing of smoking cessation and initiation of e-cigarette use. Individuals who switch to e-cigarettes may do so in response to emerging health concerns, creating a potential systematic difference between our exposure groups. While we attempted to mitigate this bias by excluding individuals with prior CVD, residual confounding may persist. Additional studies are required to fully evaluate the long-term effects of different duration and intensity of HPHC exposure due to tobacco product use and their relationship to CVD risk. Cluster analysis did not perfectly recreate the use patterns seen with concurrent survey questions, though this may reflect clustering's ability to better demonstrate real-world use patterns. Finally, our data were observational, and therefore a causal relationship cannot be drawn between clusters and CVD.

PERSPECTIVES **COMPETENCY IN MEDICAL KNOWLEDGE:** Medical Knowledge: The cluster analysis approach used in this study demonstrates the potential to objectively assess tobacco exposure patterns beyond self-reported use. Clinicians may one day use the insights from this study to guide personalized screening, counseling, and cessation interventions for patients based on their specific tobacco exposure profiles.

**TRANSLATIONAL OUTLOOK:** Additional work is needed to develop tools that can quickly translate the biomarker methodology into clinical decision support for health care providers. Exploring the utility of this approach for other tobacco and nicotine products, such as heated tobacco devices and oral nicotine pouches, will be an important next step.

## Funding support and author disclosures

This research was funded through the 10.13039/100002590American Lung Association Public Policy Research Award; 10.13039/100000050National Heart, Lung, and Blood Institute 1K01HL154130-01; and 10.13039/100000968American Heart Association Tobacco Center for Regulatory Science (Grants P50HL120163, U54HL120163, 2U54HL120163, R01HL092577) and the 10.13039/100000054National Cancer Institute and the 10.13039/100000038Food and Drug Administration Center for Tobacco Products under Award Number U54CA180905. Dr Benjamin has received support from National Heart Lung and Blood Institute (R01HL092577) and American Heart Association AF (AHA_18SFRN34110082). All other authors have reported that they have no relationships relevant to the contents of this paper to disclose.
