# Supplementary material for: Identifying Patterns of Tobacco Use and Associated Cardiovascular Disease Risk Through Machine Learning Analysis of Urine Biomarkers
Source: JACC Adv. 2025 Feb 22;4(3):101630. doi: 10.1016/j.jacadv.2025.101630 (PMC11904550; doi:10.1016/j.jacadv.2025.101630)
Supplement: Supplemental Figures 1-4, Tables 1 and 2 [file mmc1.docx]

**Supplemental** **Figure 1**: Study Inclusion/Exclusion criteria

**
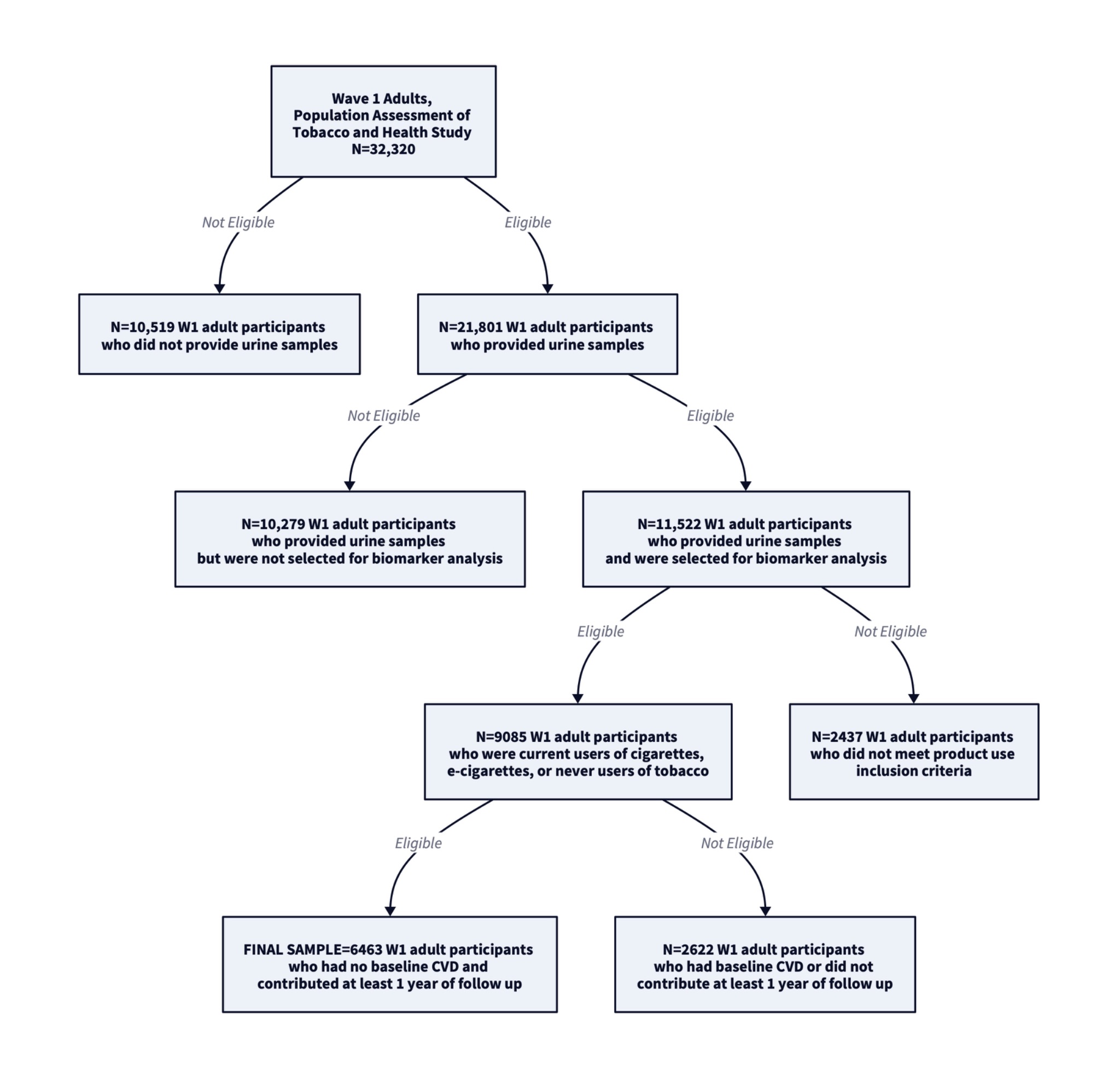
**

Supplemental Figure 1 showing that individuals included in the study were respondents to the PATH study at wave 1 who submitted a complete biomarker sample. Urine sample provision and analysis was not a function of our study team but of our PATH data source. The decision to not analyze 10,279 urine samples was a part of the PATH study statistical technique and not a decision made by our team. Individuals were included who were users of cigarettes, e-cigarettes, dual users, or never users. Individuals were only included if they had no pre-existing CVD and contributed at least 1 year of follow up.

**Supplemental** **Figure 2:** Probability of tobacco user type by cluster membership

**
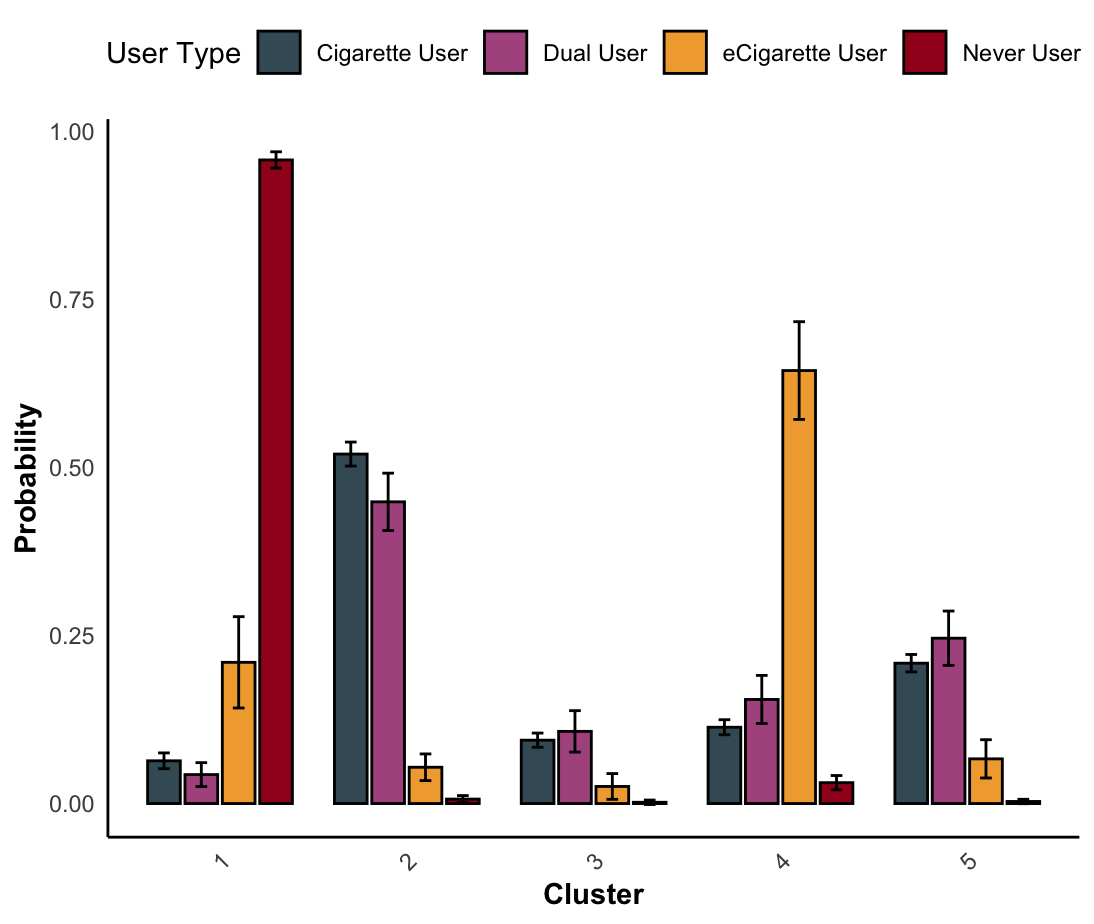
**

Supplemental Figure 2 showing probability of tobacco user type by cluster membership. Individuals in cluster 1 were significantly more likely to be non-users. Individuals in cluster 4 were more likely to be e-cigarette users. Individuals in clusters 2, 3, and 5 were more likely to be users of cigarettes or dual users.

**Supplemental** **Figure 3:** Distribution of biomarkers by cluster

**
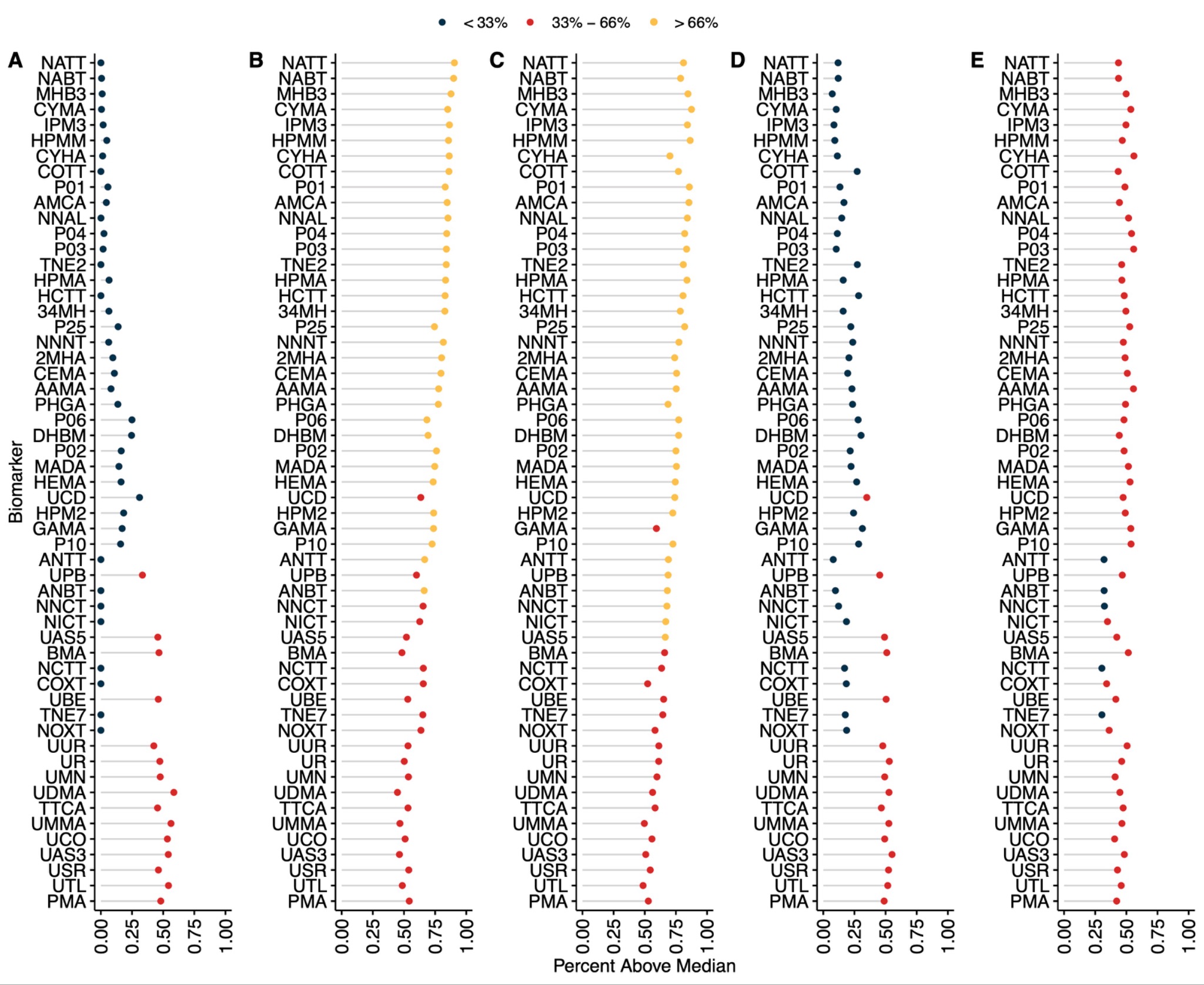
**

Supplemental Figure 3 showing distribution of biomarkers per cluster relative to total distribution of pooled biomarkers. Panel A: Biomarkers in cluster 1 were predominantly below the median of all biomarkers. Panel B: Biomarkers in cluster 4 were increased above cluster 1 but decreased below cluster 2 and located predominantly at the level of the median, or below the level of the median. Panel C: Biomarkers in clusters 3 and 5 were predominantly located at or above the level of the median although clusters 2 and 3 showed the highest levels while cluster 5 showed lower levels. Abbreviations in Table S1.

**Supplemental** **Figure 4:** Study Timeline


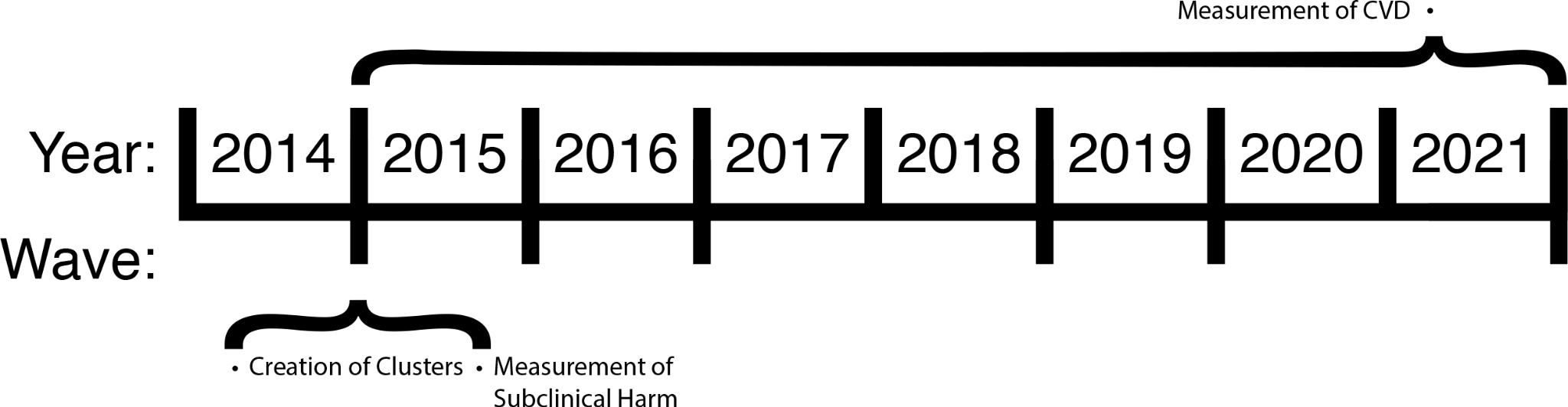


Supplemental Figure 4 showing study design and timeline. Collection of wave one was completed at 2014. Wave 1 data used to generate clusters. Wave one data was used for measurement of subclinical harm. Waves two through six were analyzed for cardiovascular outcomes.

**Supplemental** **Table 1:** Biomarkers of Exposure to Tobacco-Related Compounds and Other Chemicals

| **Abbreviation** | **Biomarker** | **Cases Imputed for clustering** | **Class** |
| --- | --- | --- | --- |
| UAS3 | Arsenous acid, urine (ug/L) | 5 | Heavy Metal |
| UAS5 | Arsenic acid, urine (ug/L) | 5 | Heavy Metal |
| UDMA | Dimethylarsinic acid, urine (ug/L) | 5 | Heavy Metal |
| UMMA | Monomethylarsonic acid, urine (ug/L) | 5 | Heavy Metal |
| UBE | Beryllium, urine (ug/L) | 20 | Heavy Metal |
| UCD | Cadmium, urine (ug/L) | 21 | Heavy Metal |
| UCO | Cobalt, urine (ug/L) | 22 | Heavy Metal |
| UMN | Manganese, urine (ug/L) | 22 | Heavy Metal |
| UPB | Lead, urine (ug/L) | 21 | Heavy Metal |
| USR | Strontium, urine (ug/L) | 32 | Heavy Metal |
| UTL | Thallium, urine (ug/L) | 21 | Heavy Metal |
| UUR | Uranium, urine (ug/L) | 22 | Heavy Metal |
| P01 | 1-Naphthol or 1-Hydroxynaphthalene (1-NAP), urine (ug/L) | 7 | Polycyclic Aromatic Hydrocarbon (PAH) |
| P02 | 2-Naphthol or 2-Hydroxynaphthalene (2-NAP), urine (ug/L) | 3 | Polycyclic Aromatic Hydrocarbon (PAH) |
| P03 | 3-Hydroxyfluorene (3-FLU), urine (ng/L) | 0 | Polycyclic Aromatic Hydrocarbon (PAH) |
| P04 | -Hydroxyfluorene (2-FLU), urine (ng/L) | 0 | Polycyclic Aromatic Hydrocarbon (PAH) |
| P06 | 1-Hydroxyphenanthrene (1-PHE), urine (ng/L): | 0 | Polycyclic Aromatic Hydrocarbon (PAH) |
| P10 | 1-Hydroxypyrene (1-PYR), urine (ng/L) | 0 | Polycyclic Aromatic Hydrocarbon (PAH) |
| P25 | 2-Hydroxyphenanthrene and 3-Hydroxyphenanthrene (2-3PHE), urine (ng/L) | 0 | Polycyclic Aromatic Hydrocarbon (PAH) |
| NABT | N'-Nitrosoanabasine (NAB), urine (ng/mL) | 21 | Nicotine Metabolite |
| NATT | N'-Nitrosoanatabine (NAT), urine (ng/mL) | 55 | Nicotine Metabolite |
| NNAL | 4-(methylnitrosamino)-1-(3-pyridyl)-1-butanol (NNAL), urine (ng/mL) | 7 | Nicotine Metabolite |
| NNNT | N'-Nitrosonornicotine (NNN), urine (ng/mL) | 142 | Nicotine Metabolite |
| **Abbreviation** | **Biomarker** | **Cases Imputed for clustering** | **Class** |
| ANBT | Anabasine, urine (ng/mL) | 1,621 | Nicotine Metabolite |
| ANTT | Anatabine, urine (ng/mL) | 1,625 | Nicotine Metabolite |
| COTT | Cotinine, urine (ng/mL) | 10 | Nicotine Metabolite |
| COXT | Cotinine N-oxide, urine (ng/mL) | 1,621 | Nicotine Metabolite |
| HCTT | trans-3'-Hydroxycotinine, urine (ng/mL) | 17 | Nicotine Metabolite |
| NCTT | Norcotinine, urine (ng/mL) | 1,621 | Nicotine Metabolite |
| NICT | Nicotine, urine (ng/mL) | 1,621 | Nicotine Metabolite |
| NNCT | Nornicotine, urine (ng/mL) | 1,630 | Nicotine Metabolite |
| NOXT | Nicotine N-oxide, urine (ng/mL) | 1,621 | Nicotine Metabolite |
| TNE2 | Total Nicotine Equivalents (2): The molar sum of the imputed values of Cotinine, and Trans-3'-Hydroxycotinine, urine (nmol/mL) | 24 | Nicotine Metabolite |
| TNE7 | Total Nicotine Equivalents (7): The molar sum of the imputed values of Cotinine, Trans-3'-Hydroxycotinine, Cotinine N-oxide, Nicotine N-oxide, Norcotinine, Nornicotine, and Nicotine, urine (nmol/mL) | 1,630 | Nicotine Metabolite |
| UR | Molar Ratio of the imputed values of Trans-3'-Hydroxycotinine to Cotinine in urine | 24 | Nicotine Metabolite |
| 2MHA | 2-methylhippuric acid | 196 | Volatile Organic Compounds (VOC) |
| 34MH | 3- and 4-methylhippuric acids | 4 | Volatile Organic Compounds (VOC) |
| AAMA | N-Acetyl-S-(2-carbamoylethyl)-L-cysteine | 49 | Volatile Organic Compounds (VOC) |
| AMCA | N-Acetyl-S-(N-methylcarbamoyl)-L-cysteine | 44 | Volatile Organic Compounds (VOC) |
| BMA | N-Acetyl-S-(benzyl)-L-cysteine | 23 | Volatile Organic Compounds (VOC) |
| CEMA | N-Acetyl-S-(2-carboxyethyl)-L-cysteine | 324 | Volatile Organic Compounds (VOC) |
| **Abbreviation** | **Biomarker** | **Cases Imputed for clustering** | **Class** |
| CYHA | N-Acetyl-S-(1-cyano-2-hydroxyethyl)-L-cysteine | 1 | Volatile Organic Compounds (VOC) |
| CYMA | N-Acetyl-S-(2-cyanoethyl)-L-cysteine | 1 |  |
| DHBM | N-Acetyl-S-(3,4-dihydroxybutyl)-L-cysteine | 599 | Volatile Organic Compounds (VOC) |
| GAMA | N-Acetyl-S-(2-carbamoyl-2-hydroxyethyl)-L-cysteine | 374 | Volatile Organic Compounds (VOC) |
| HEMA | N-Acetyl-S-(2-hydroxyethyl)-L-cysteine | 720 | Volatile Organic Compounds (VOC) |
| HPM2 | N-Acetyl-S-(2-hydroxypropyl)-L-cysteine | 79 | Volatile Organic Compounds (VOC) |
| HPMA | N-Acetyl-S-(3-hydroxypropyl)-L-cysteine | 63 | Volatile Organic Compounds (VOC) |
| HPMM | N-Acetyl-S-(3-hydroxypropyl-1-methyl)-L-cysteine | 1 | Volatile Organic Compounds (VOC) |
| IPM3 | N-Acetyl-S-(4-hydroxy-2-methyl-2-buten-1-yl)-L-cysteine | 30 | Volatile Organic Compounds (VOC) |
| MADA | Mandelic acid | 536 | Volatile Organic Compounds (VOC) |
| MHB3 | N-Acetyl-S-(4-hydroxy-2-buten-1-yl)-L-cysteine | 1 | Volatile Organic Compounds (VOC) |
| PHGA | Phenylglyoxylic acid | 300 | Volatile Organic Compounds (VOC) |
| PMA | N-Acetyl-S-(phenyl)-L-cysteine | 1 | Volatile Organic Compounds (VOC) |
| TTCA | 2-Thioxothiazolidine-4-carboxylic acid | 758 | Volatile Organic Compounds (VOC) |
| IL6 | Interleukin 6 | NA | Inflammatory Marker |
| SICAM | Soluble intercellular adhesion molecule | NA | Inflammatory Marker |
| 8PGFT | 8-isoprostane | NA | Inflammatory Marker |
| hsCRP | High Sensitivity C Reactive Protein | NA | Inflammatory Marker |

Supplemental Table 1 showing abbreviations of all biomarkers used throughout the text and figures, as well as cases imputed for clustering. Cases were only imputed for clustering purposes. For all other analyses, data was analyzed excluding missing cases. Abbreviations used throughout paper to represent chemical or value. The biomarker column identifies the chemical in common scientific name or explanation of value. Class column describes subclasses of chemicals relevant to tobacco research.

**Supplemental** **Table 2:** Bayesian Information Criteria of Cluster Labelings with Varying Numbers of Clusters

| **Number of Clusters** | **BIC** |
| --- | --- |
| 1 | 536,159.65 |
| 2 | 464,444.03 |
| 3 | 307,514.63 |
| 4 | 275,854.80 |
| 5 | 228,525.02 |
| 6 | 230495.4898 |
| 7 | 241723.0669 |
| 8 | 239652.2885 |
| 9 | 250821.9761 |
| 10 | 250211.5838 |
| 11 | 272617.4179 |

Supplemental Table 2 shows that BIC decreases with additions of clusters until it reaches a global minimum at 5 clusters. After 5 clusters, BIC continues to increase with each additional cluster.
